# Europe PMC in 2017

**DOI:** 10.1093/nar/gkx1005

**Published:** 2017-11-17

**Authors:** Maria Levchenko, Yuci Gou, Florian Graef, Audrey Hamelers, Zhan Huang, Michele Ide-Smith, Anusha Iyer, Oliver Kilian, Jyothi Katuri, Jee-Hyub Kim, Nikos Marinos, Rakesh Nambiar, Michael Parkin, Xingjun Pi, Frances Rogers, Francesco Talo, Vid Vartak, Aravind Venkatesan, Johanna McEntyre

**Affiliations:** European Molecular Biology Laboratory, European Bioinformatics Institute (EMBL-EBI), Wellcome Trust Genome Campus, Cambridge, UK

## Abstract

Europe PMC (https://europepmc.org) is a comprehensive resource of biomedical research publications that offers advanced tools for search, retrieval, and interaction with the scientific literature. This article outlines new developments since 2014. In addition to delivering the core database and services, Europe PMC focuses on three areas of development: individual user services, data integration, and infrastructure to support text and data mining. Europe PMC now provides user accounts to save search queries and claim publications to ORCIDs, as well as open access profiles for authors based on public ORCID records. We continue to foster connections between scientific data and literature in a number of ways. All the data behind the paper - whether in structured archives, generic archives or as supplemental files - are now available via links to the BioStudies database. Text-mined biological concepts, including database accession numbers and data DOIs, are highlighted in the text and linked to the appropriate data resources. The SciLite community annotation platform accepts text-mining results from various contributors and overlays them on research articles as licence allows. In addition, text miners and developers can access all open content via APIs or via the FTP site.

## BACKGROUND

The core mission of Europe PMC is to build open, full text scientific literature resources and support innovation by engaging users, enabling contributors and integrating related research data. Europe PMC is an ELIXIR Core Data Resource, selected among other European data resources of fundamental importance to the wider life-sciences community and the long-term preservation of biological data (https://www.elixir-europe.org/platforms/data/core-data-resources) (1). Formerly known as UKPMC (2), the service was rebranded in November 2012 as Europe PMC (https://europepmc.org) (3) to reflect the Europe-wide funders that support it. Since then, the funders group geography has expanded beyond Europe and currently Europe PMC is the designated repository for the open access publication mandates of 28 life sciences funding organisations, including international agencies, such as the European Research Council (ERC) and the World Health Organisation (WHO). Researchers funded by these organisations can deposit manuscripts and link publications to grants using the Europe PMC Plus submission system (https://plus.europepmc.org).

The content scope of Europe PMC covers both abstracts and full text articles. As a partner in PMC International (PMCi), along with PMC USA and PMC Canada (https://www.ncbi.nlm.nih.gov/pmc/about/pmci), Europe PMC shares full text content with all the members of this initiative. In addition to the full text collection, Europe PMC provides access to all PubMed abstracts, selected Agricola abstracts (https://agricola.nal.usda.gov/), international patents from the European Patent Office (https://www.epo.org), as well as documents from several other sources. Altogether, Europe PMC combines ∼33 million abstracts and 4.3 million full text articles in a single search interface.

Along with the publications, Europe PMC operates a citation network, determined from the Europe PMC content and supplemented with metadata supplied by CrossRef (https://www.crossref.org). This enables features like citation counts sort order, ‘Cited By’ lists for articles referencing a particular publication, and citation graphs for individual papers, showing how frequently they have been cited over time.

To facilitate information discovery and foster literature–data integration, Europe PMC has incorporated text-mining approaches into its workflows. The text-mined terms and data links can be explored via the BioEntities tab on the website, or as highlights in the text using the SciLite annotations function (see sections Data Integration and Resources for Text and Data Miners for more details) (4). The External Links service (https://europepmc.org/LabsLink) provides additional literature–data connections. It enhances existing content and helps users find relevant additional resources, such as alternative metrics, peer reviews, the full text of publications in institutional repositories, Wikipedia articles, or teaching materials.

We strive to provide a world-class, reliable and high-performance service. To ensure this objective, we have made a number of general improvements concerning our technical infrastructure and processes. For example, we now serve abstracts from MongoDB (a document-based database) for rapid retrieval of documents and text-mined annotations. The migration of indexing services from Lucene to Solr has allowed us to make use of out-of-the-box capabilities such as faceting for the new date filter on the search results page (see section Access to Content). In addition to such infrastructural changes, we are committed to improving the user experience of Europe PMC by understanding the needs of key stakeholder groups, such as life sciences researchers, curators, developers and text miners. We follow a user-centered design process based on user research involving many participants worldwide with varied backgrounds and use cases. The outcomes of the user research programme inform and guide the development of the website and help us understand whether new features are adopted by users and how they are being utilised. Some highlights of a user research study of literature search behaviour can be found in the Europe PMC Literature Search User Research Report (https://doi.org/10.6084/m9.figshare.4789744.v1).

In the interests of transparency for all our users around upcoming developments, we publish a Europe PMC roadmap (https://europepmc.org/Roadmap) that shows work in progress, as well as future development priorities. Europe PMC community outreach programme aims to engage the key stakeholder groups to build a community of users. We therefore welcome all feedback on any aspect of the service at helpdesk@europepmc.org.

## ACCESS TO CONTENT

### Programmatic users

Europe PMC provides several tools and services to access the data. All of the content in Europe PMC is free to read, however only a proportion is available for reuse, for purposes such as translation or text and data mining. Europe PMC maximises the potential for reuse by sharing bulk content in the following ways.

#### Webservices

Europe PMC offers RESTful and SOAP webservices (https://europepmc.org/developers) for access to abstracts, full text articles and grants, as well as additional content such as text-mined annotations, ORCIDs, and links to data. The RESTful API has a modular structure and outputs in XML, JSON or Dublin Core formats. Most recently, we have developed an annotations API from which any annotation of Europe PMC content can be retrieved, including community annotations from third parties (see sections Data Integration and Resources for Text and Data Miners for more details).

#### Bulk download

The full text open access articles in Europe PMC (∼1.7 million at the time of writing) are available for bulk download on the Europe PMC FTP site (https://europepmc.org/downloads) and are served in full using Europe PMC RESTful and SOAP APIs. Other Europe PMC content available for bulk download includes the author manuscript collection (around 600K accepted manuscripts that were uploaded by authors to PubMed Central and Europe PMC), the metadata from all full text Europe PMC articles, supplementary data files, DOI–PMCID–PMID mappings and text-mined accession numbers from major life sciences databases.

### Website users

Europe PMC offers a unified search for all the content, including both abstracts and articles full text. Users can create complex, customised queries using advanced search functions. To deliver novel full text search and viewing mechanisms we implement continuous improvements to the service. Some of these are highlighted below.

#### Author search

**Figure 1.**
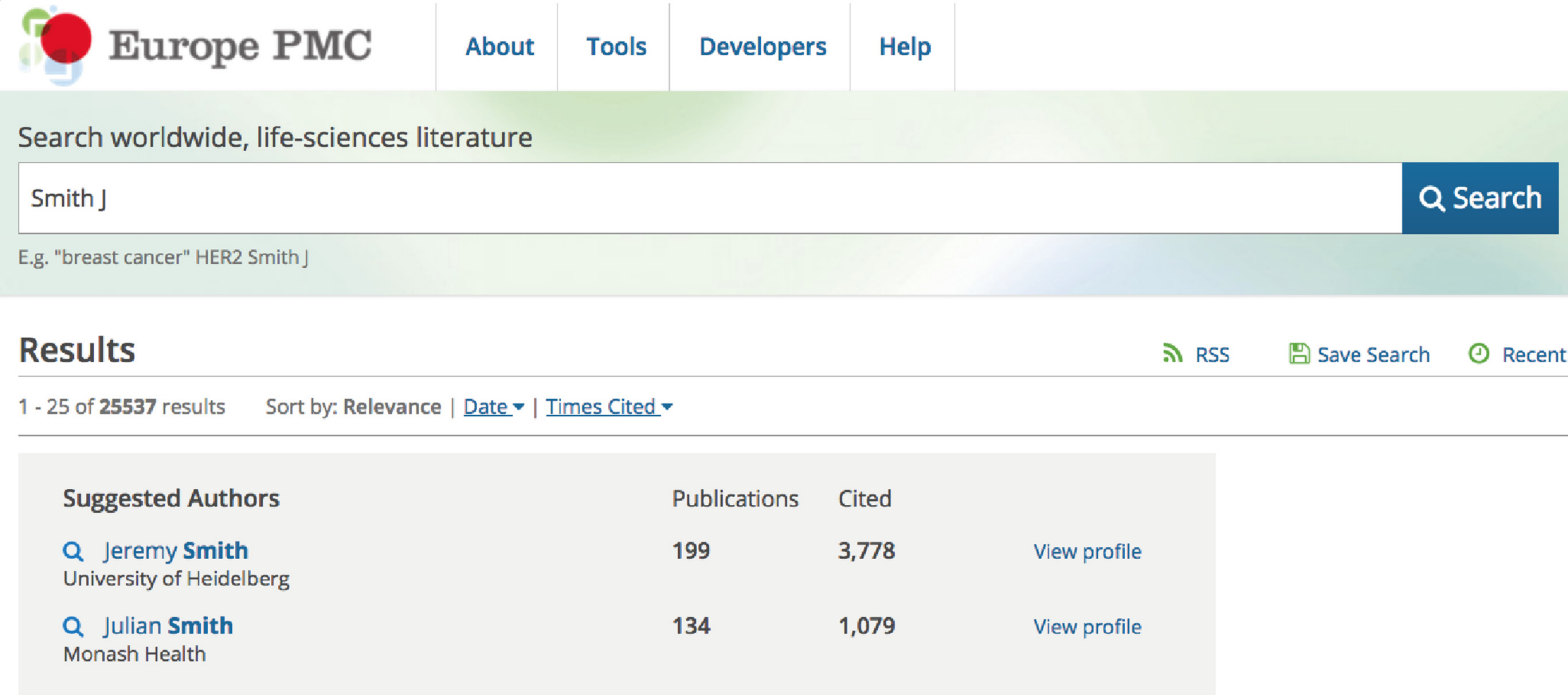
Searching for authors in Europe PMC. If the name of the author in the search query matches a name on an ORCID record in Europe PMC, the user will see a ‘Suggested Authors’ box on top of their results list. The box will provide additional information about the author, such as affiliation (if available), number of publications and citations, to help the user decide if this was the researcher they were looking for.

**Figure 2.**
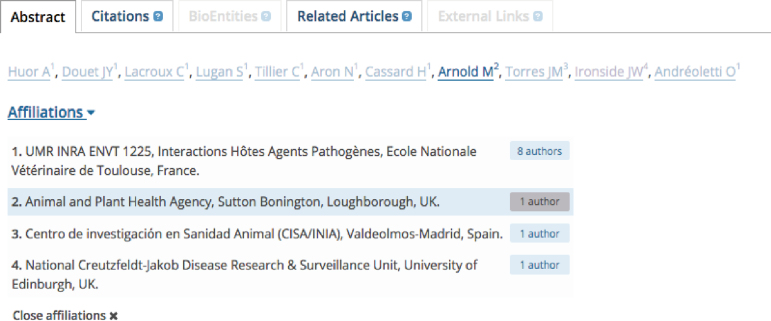
Affiliation information display. Affiliation information can be found on the publication abstract under the list of authors. Users can select an individual affiliation, which then highlights the names of the authors affiliated with that particular institution.

To help disambiguate academic authors, Europe PMC has previously integrated ORCIDs (https://orcid.org), unique researcher identifiers. In addition to ORCID-based author searching, Europe PMC now provides a ‘Suggested Authors’ feature. When searching for an author by name, which matches a researcher with an ORCID, the search will bring up a box with suggested authors at the top of the results list. Europe PMC will display up to two matching researchers ranked by number of publications in their profile (Figure 1). We have also improved the author affiliation display on the abstract pages. Affiliation information can be found under an expandable link, and clicking on an individual institution highlights the names of the researchers in the authors list that are affiliated with it (Figure 2). Note that the affiliation information displayed in Europe PMC is supplied by those submitting the manuscript, and institution names are not disambiguated at present.

#### Date search

While sophisticated date searching can be specified via the Advanced Search page, a publication date facet on the search results page automatically subsets the results by publication year, making it easy to spot trends or limit the search to a particular range of years. Furthermore, when the full text of articles is under embargo from the publisher, the release date now appears on the abstract page of a given publication to inform readers when the full text will become available in Europe PMC.

#### Grant search

The Europe PMC website now provides a redesigned Grant Finder tool (https://europepmc.org/grantfinder). The new interface uses the public GRIST API (https://europepmc.org/GristAPI) and allows searching and browsing of grant data, including links to associated publications. With the help of the Grant Finder you can find the details for over 60,000 biomedical research grants, awarded to the 28 000 researchers supported by the 28 Europe PMC funders. The tool enables you to search for grants by funder, principal investigator name, ORCID, affiliation, as well as by keyword in the grant title, abstract, funding stream and grant type. In addition to the Grant Finder, grant information from funding organisations beyond Europe PMC funders group can be explored using Funding Attribution as the advanced search query parameter. The funding agency name and the grant number associated with a particular publication are displayed on the article abstract page.

## INDIVIDUAL USER SERVICES

### Author profiles

**Figure 3.**
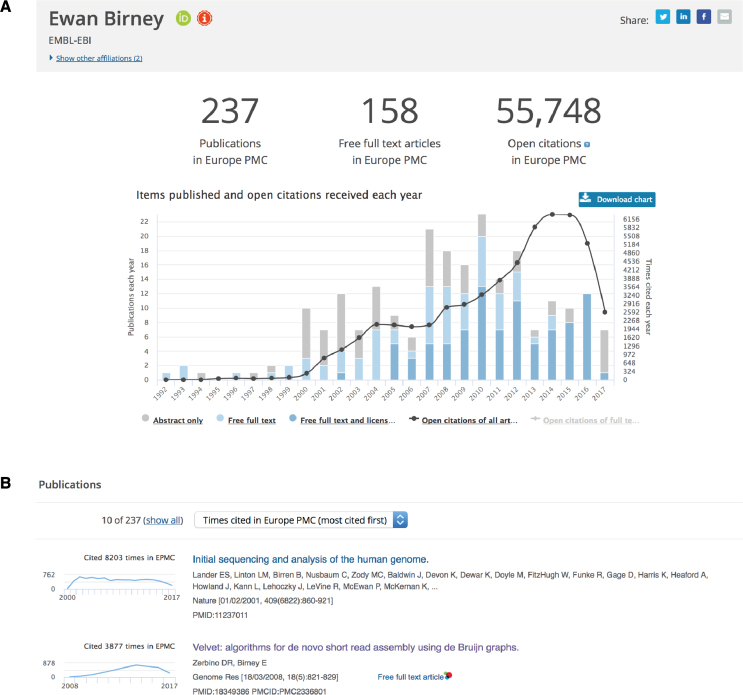
Author profiles in Europe PMC. An author profile showcases published works of a given author with an ORCID in Europe PMC. (**A**) The publication graph combines a bar chart, representing the number of publications per year, and a line graph, which shows the number of citations per year. Open access publications are highlighted in blue, with articles for which free full text is available depicted in light blue, and articles licensed for reuse represented with a darker shade. (**B**) The publication list includes all articles in Europe PMC claimed to the author's ORCID record. For any given publication we display citation counts along with a graph showing citation count per year. The list can be sorted by ‘date’ or ‘times cited’.

**Figure 4.**
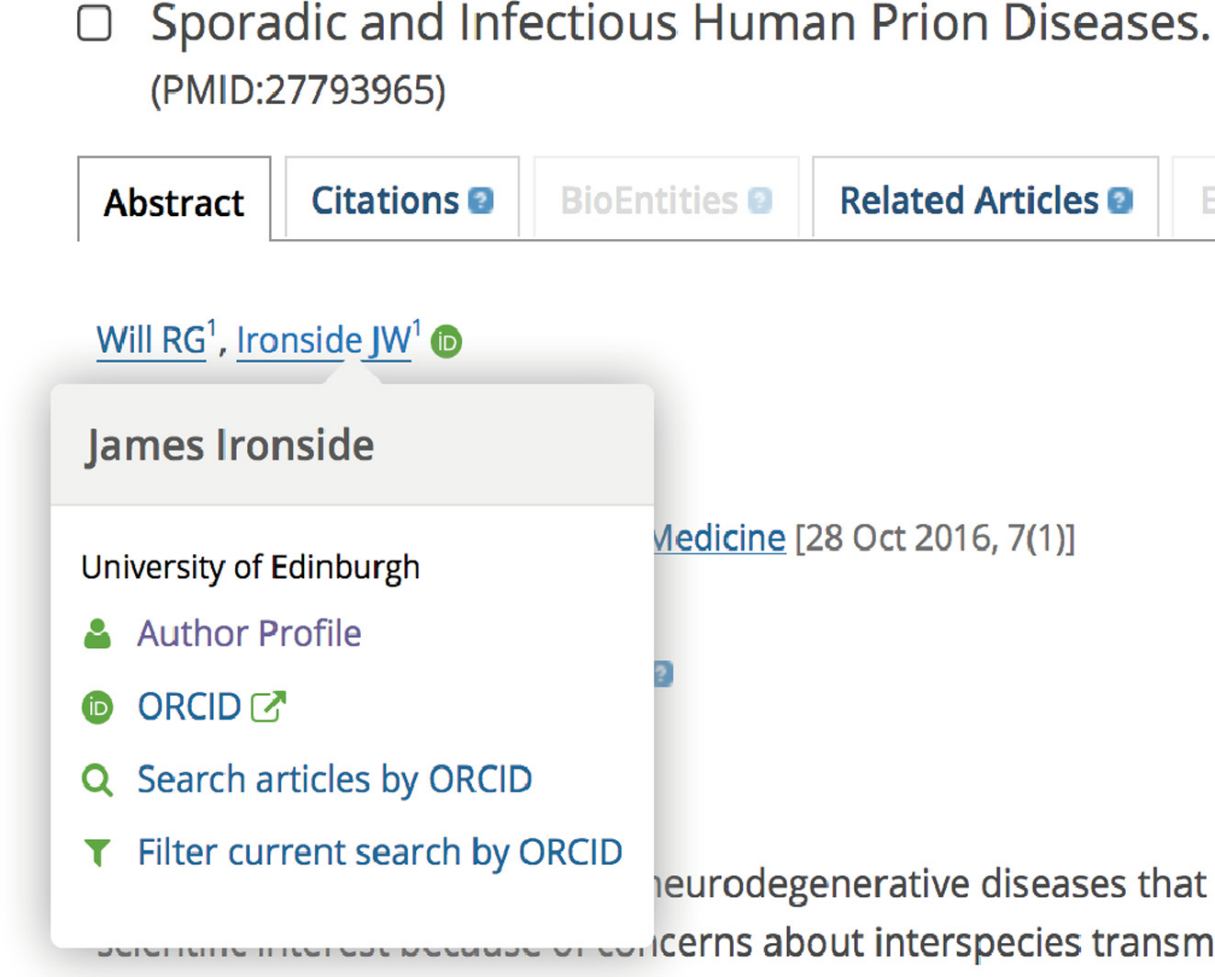
Author profile links on abstract pages in Europe PMC. Users will see a popup menu when clicking on the author's name in the list. The menu will contain a link to the author's profile and ORCID record, along with additional search options.

To support individual researchers, we aim to provide useful tools for various scientific workflows. The most notable new development in this regard is the launch of the author profiles. The author profiles are automatically generated for any author with a public ORCID record, who has works available in Europe PMC. With a growing number of articles linked to ORCIDs (currently ∼4.5 million in Europe PMC linked to more than 500 000 unique ORCIDs), this feature will be of increasing interest to authors, publishers, funders, and others interested in scientific credit. An author profile provides a graphical overview of the author's academic output. A publication graph displays author's works in Europe PMC for any given year, as well as an indication of the proportion of open access articles (Figure 3A). Each article is also listed individually with a citation graph showing the citation count over time (Figure 3B). Similar graphs can be seen on full text and abstract pages for other publications in Europe PMC. The list is automatically updated whenever a user claims a publication to their ORCID, or if the publication is added to their ORCID on their behalf, for example by a publisher. A personal profile can be found via the following link: https://europepmc.org/authors/0000-0000-0000-0000, where 0000–0000-0000–0000 is your ORCID. The link to an author profile appears following an author search, or on the abstract pages of individual publications (Figure 4).

### User accounts

**Figure 5.**
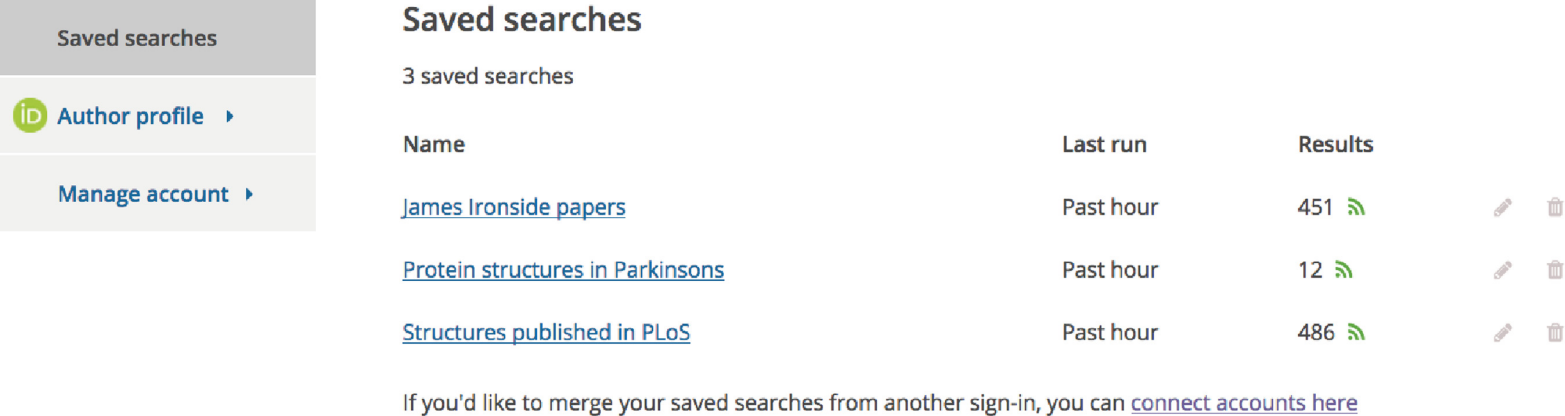
User accounts in Europe PMC. Users can sign into Europe PMC to save their searches. To create an alert about new publications matching a query it is possible to set up a customised RSS feed based on a saved search by clicking on the RSS icon next to the search results.

Europe PMC users can now create and use an account to save and manage searches, claim publications to their ORCID record or submit author manuscripts via the Europe PMC Plus system (for researchers supported by Europe PMC funders) (Figure 5). The saved searches feature assists with running frequent queries and allows the user to receive automatic updates for their search by setting up a user-defined RSS feed based on the saved query.

## DATA INTEGRATION

At Europe PMC it is part of our mission to create solutions that make it easier for scientists to navigate the data-rich literature in the most efficient way and to provide feedback on data citation to data resources.

### Links to associated data

For many biological studies, it is common nowadays to produce several types of data that may reside in different databases, or as supplementary files appended to articles. To address the challenge of linking all the data pertaining to a study together, the EMBL-EBI has developed BioStudies (https://www.ebi.ac.uk/biostudies/), a new database that acts as a data container and holds descriptions of biological studies and links to data from these studies in other databases, while archiving data that do not fit in the structured archives (5). BioStudies provides a permanent location for Europe PMC supplemental data, making it citable and linking it to any other data from the study residing in public repositories, which makes it more available for re-use and discovery. BioStudies is especially valuable for multi-omics experiments, where different types of data can be produced, or for large studies, where more than one paper is published on a single dataset, which then becomes a single point of reference as a BioStudies record. For every full text article in Europe PMC that either has supplemental data files or mentions data accession numbers (identified by text-mining), a BioStudies record is generated and linked to the article. The Europe PMC text‐mining pipeline (6) covers 20 major data resources in the life sciences, including ArrayExpress, BioProject, BioSample, ClinicalTrials.gov, EudraCT, EGA, EMDB, Ensembl, ENA, GO, InterPro, OMIM, PDBe, Pfam, ProteomeXchange, RefSeq, RefSNP, TreeFam, UniProt, as well as data DOIs. Over a million Europe PMC articles now have corresponding BioStudies records, which can be explored by clicking on the BioStudies link on the abstract pages.

### SciLite annotations

In 2016 we developed SciLite (https://europepmc.org/Annotations)—a tool that allows biological terms or functional relationships to be highlighted in the text (4). These concepts are identified by text-mining algorithms, developed by a number of groups from the text-mining community. Annotations appear on all abstracts, and full text articles with a CC-BY, CC-BY-NC or CC0 license (around 1 million articles at the time of writing). SciLite can assist skim-reading of articles, extracting facts and evidence from the scientific literature, as well as locating the primary data, such as nucleotide sequences or protein structures, cited in a given publication.

**Figure 6.**
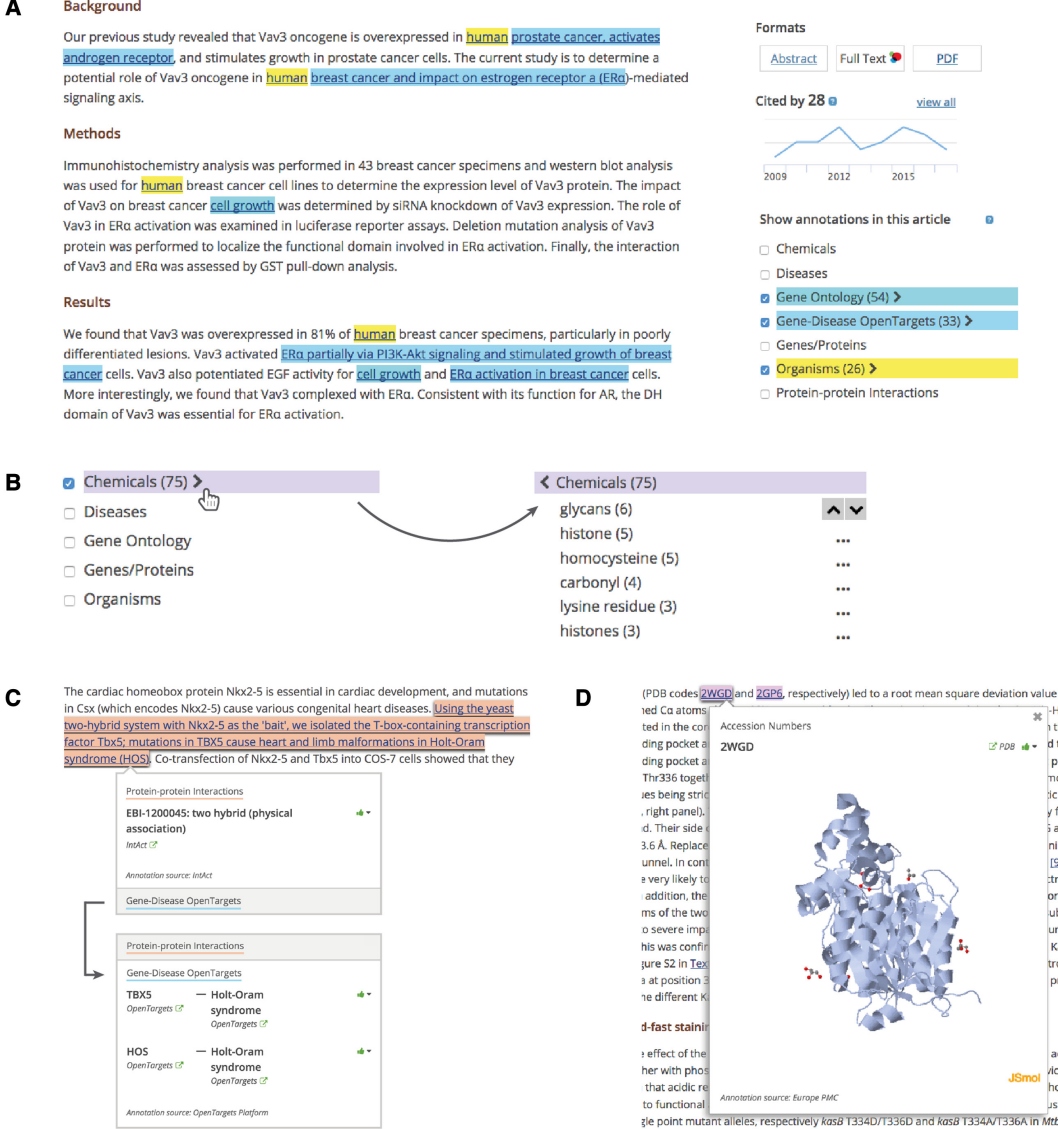
SciLite annotations in Europe PMC. Text-mined terms can be highlighted in the publication text using the SciLite annotations tool. (**A**) The annotations menu enables the user to highlight a selection of colour-coded annotations in the text (PMCID:PMC2430719). (**B**) To see a list of individual terms, users may click on the right arrow next to the annotation type. A selection of terms found most frequently in the text appears, together with up/down navigation buttons, which allow the user to jump to selected terms in the text. (**C**) An entire sentence is highlighted for gene–disease associations and protein–protein interactions. In case of overlapping annotations it is possible to view the annotation types appearing under the same highlight by selecting the annotation of interest in the popup box (PMID:11431700). (**D**) Clicking on the highlighted terms in the text opens a popup box with information about the given annotation, including a link to related database record, the source of the annotation, and the feedback link. In some cases, the popup will also contain a visual data preview, for example the menu for the annotated Protein Data Bank accession number will display an interactive thumbnail of the macromolecular structure (PMCID:PMC4014462).

SciLite annotations include core named entities (gene/protein names, organisms, diseases, chemicals, Gene Ontology terms, etc.), biological events (phosphorylation), functional relations (gene–disease associations, protein–protein interactions), as well as biological functions (gene function). When viewing an article, any annotations can be explored in an interactive menu alongside the publication (Figure 6A). A selection of individual terms found most frequently in the text is available for most annotation types (Figure 6B). For some annotation types, such as gene–disease associations and protein–protein interactions, an entire sentence that contains the evidence for the functional relation is highlighted. It is possible for a sentence to contain overlapping annotations. In such a case, the reader can see individual annotation types within a popup box that appears when clicking on the highlight (Figure 6C).

To foster connections between scientific literature and data, individual annotations are linked to corresponding records in public databases. Those links can be found in a popup box displayed when a reader clicks on the highlighted term (Figure 6D). In addition, SciLite highlights data citations in the form of text-mined accession numbers, which help locate the underlying data record mentioned in the text.

It is of critical importance that readers find the annotations useful. Readers can validate individual annotations, providing feedback that can improve text-mining accuracy. The feedback link appears on the popup window, allowing users to endorse useful annotations, or report incorrect or generic annotations. The reported incorrect annotations are automatically removed within 24 h, and the feedback is collected and reviewed to improve the text-mining algorithms. This type of community feedback is the main mechanism for quality control of the text-mining outputs displayed by the SciLite application.

## RESOURCES FOR TEXT AND DATA MINING

One of the goals of open access is to stimulate innovation: to discover and use the content in new ways. To support such advances through text and data mining, Europe PMC operates a community annotations platform, which provides the means for text miners to access content and publish the results of text-mining as annotations on papers, giving exposure of their work to a wider audience (https://europepmc.org/Annotations#info-for-text-miners). The platform accepts outputs from any contributor, and makes them available to users both programmatically, via the API in JSON format (https://europepmc.org/AnnotationsApi), and on the website with the help of the SciLite application. Received annotations are modelled according to the W3C standard Web Annotation Data Model. The open nature and standard format of the SciLite annotations means that they can be shared, reused and potentially integrated with other types of annotation, such as comments. Annotations are exported to a triple store as RDF, which provides an opportunity to explore the annotations in conjunction with other RDF graphs in the Linked Data cloud. The SciLite RDF endpoint is publicly available (http://www.ebi.ac.uk/europepmc/rdf/sparql) and contains over 1.4 billion triples (at the time of writing). Any interested text-mining group that would like to supply annotations to the platform is welcome to contact us at helpdesk@europepmc.org.

## SUMMARY

Europe PMC is a fast, reliable and comprehensive resource for accessing the life sciences literature based on open access and open data principles. The goal is to establish Europe PMC as an innovation platform, open for new developments coming from the community itself. Therefore, our community engagement efforts are central to building the platform. Viewing the literature as a bridging mechanism for wider research infrastructure, we focus on linking literature and data in order to support complex researcher workflows that rely on finding and retrieving supporting data quickly and easily.

We welcome comments and questions about any aspect of the service. The best way to contact us is via the Feedback button at the bottom of every page. Queries about working with Europe PMC webservices can be directed to the public developer forum (https://groups.google.com/a/ebi.ac.uk/forum/#!forum/epmc-webservices). We also provide external user support with online training covering different aspects of the Europe PMC service, which is available through the EMBL-EBI Train Online interface (https://www.ebi.ac.uk/training/online/course-subject-area/literature). We are active on Twitter (https://twitter.com/EuropePMC_news) and you can follow @EuropePMC_news for updates about the Europe PMC service. New developments are routinely outlined on the Europe PMC blog (http://blog.europepmc.org/).

## References

[1] Durinx C., McEntyre J., Appel R., Apweiler R., Barlow M., Blomberg N., Cook C., Gasteiger E., Kim J.H., Lopez R. Identifying ELIXIR Core Data Resources [version 2; referees: 2 approved]. F1000Research. 2017; 5:2422. 10.12688/f1000research.9656.1 PMC5070591 27803796

[2] McEntyre J.R., Ananiadou S., Andrews S., Black W.J., Boulderstone R., Buttery P., Chaplin D., Chevuru S., Cobley N., Coleman L.A. UKPMC: a full text article resource for the life sciences. Nucleic Acids Res. 2011; 39:D58–D65. 21062818 10.1093/nar/gkq1063 PMC3013671

[3] The Europe PMC Consortium. Europe PMC: a full-text literature database for the life sciences and platform for innovation. Nucleic Acids Res. 2015; 43:D1042–D1048. 25378340 10.1093/nar/gku1061 PMC4383902

[4] Venkatesan A., Kim J.H., Talo F., Ide-Smith M., Gobeill J., Carter J., Batista-Navarro R., Ananiadou S., Ruch P., McEntyre J. SciLite: a platform for displaying text-mined annotations as a means to link research articles with biological data [version 2; referees: 2 approved, 1 approved with reservations]. Wellcome Open Res. 2017; 1:25. 28948232 10.12688/wellcomeopenres.10210.2 PMC5527546

[5] McEntyre J., Sarkans U., Brazma A. The BioStudies database. Mol. Syst. Biol. 2015; 11:847. 26700850 10.15252/msb.20156658 PMC4704487

[6] Kafkas S., Kim J.H., McEntyre J. Database citation in full text biomedical research articles. PLoS ONE. 2013; 8:e63184. 23734176 10.1371/journal.pone.0063184 PMC3667078

